# Identification of a Novel Chromosomal Passenger Complex and Its Unique Localization during Cytokinesis in *Trypanosoma brucei*

**DOI:** 10.1371/journal.pone.0002354

**Published:** 2008-06-11

**Authors:** Ziyin Li, Ju Huck Lee, Feixia Chu, Alma L. Burlingame, Arthur Günzl, Ching C. Wang

**Affiliations:** 1 Department of Pharmaceutical Chemistry, University of California San Francisco, San Francisco, California, United States of America; 2 Department of Genetics and Developmental Biology, University of Connecticut Health Center, Farmington, Connecticut, United States of America; New England Biolabs, United States of America

## Abstract

Aurora B kinase is a key component of the chromosomal passenger complex (CPC), which regulates chromosome segregation and cytokinesis. An ortholog of Aurora B was characterized in *Trypanosoma brucei* (TbAUK1), but other conserved components of the complex have not been found. Here we identified four novel TbAUK1 associated proteins by tandem affinity purification and mass spectrometry. Among these four proteins, TbKIN-A and TbKIN-B are novel kinesin homologs, whereas TbCPC1 and TbCPC2 are hypothetical proteins without any sequence similarity to those known CPC components from yeasts and metazoans. RNAi-mediated silencing of each of the four genes led to loss of spindle assembly, chromosome segregation and cytokinesis. TbKIN-A localizes to the mitotic spindle and TbKIN-B to the spindle midzone during mitosis, whereas TbCPC1, TbCPC2 and TbAUK1 display the dynamic localization pattern of a CPC. After mitosis, the CPC disappears from the central spindle and re-localizes at a dorsal mid-point of the mother cell, where the anterior tip of the daughter cell is tethered, to start cell division toward the posterior end, indicating a most unusual CPC-initiated cytokinesis in a eukaryote.

## Introduction

*Trypanosoma brucei* is an ancient protozoan parasite and the causative pathogen of sleeping sickness in human and nagana in various livestock in sub-Saharan Africa. The cell cycle of *T. brucei* displays several unique features (for a review, see [1]): firstly, trypanosomes possess a single mitochondrion with a distinct division cycle [2]. Thus, a well-regulated coordination between mitochondrial and nuclear cycles is essential for successful cell division [3]. Secondly, cytokinesis in *T. brucei* depends primarily on the segregation of a replicated flagellar basal body, which is vastly different from that in higher eukaryotes [4], [5]. The components of cleavage furrow have not been identified in trypanosomes, and an actomyosin contractile ring, usually observed in the cleavage furrows of yeasts and higher eukaryotes, is not found in trypanosomes [6]. Cytokinesis in the insect (procyclic) form of *T. brucei* is initiated at the anterior end of the daughter cell tethered to the dorsal mid-point of mother cell and proceeds in a helical manner following the new flagellum/flagellum attachment zone (FAZ) as axis [5], [7]. Thirdly, there are differences in cell cycle regulation between procyclic and bloodstream trypanosomes. Inhibition of mitosis in the procyclic form does not totally block cytokinesis resulting in anucleate daughter cells with single mitochondrial DNA complexes (kinetoplasts) [3], [8], [9]. In contrast, inhibition of mitosis in bloodstream cells prevents cytokinesis, but allows continuous progression of the nuclear cycle and organelle replication thus producing giant polyploid cells with multiple kinetoplasts, basal bodies and flagella [9]–[11]. Exploration of these unique features in *T. brucei* may facilitate our understanding of the molecular mechanisms which coordinate the nuclear and kinetoplast cycles, initiate cytokinesis in two different ways between two developmental stages, and drive a novel mode of cell division in a deeply-branching eukaryote.

One of the effective approaches to dissect these interesting phenomena has been to characterize a protein in *T. brucei* whose ortholog in other eukaryotes is known to play essential roles in controlling both mitosis and cytokinesis. Aurora B kinase is such a protein because it is a key component of the chromosomal passenger complex (CPC) required for bipolar spindle assembly, chromosome segregation and cytokinesis in eukaryotes (for recent reviews, see [12], [13]). Metazoan CPC contains three additional non-enzymatic proteins INCENP, Survivin, and Borealin/Dasra-B which are well conserved and play essential roles for the function of Aurora B. Yeasts have orthologs of Aurora B, INCENP and Survivin but not Borealin [13]. CPC localization during mitosis is highly dynamic. Being closely associated with chromatin before the onset of mitosis, they move from the chromosome arms toward the inner centromeric chromatin during prometaphase, reappear on the microtubules of the central spindle at the metaphase-anaphase transition, and finally concentrate in the spindle midzone during telophase [12]. When cytokinesis starts, CPC merges with the cleavage furrow made of an actomyosin contractile ring upon dissolution of the nuclear envelope [14].

Aurora B phosphorylates its substrates and regulating their localizations and/or activities. While associated with chromatin, Aurora B regulates chromatin condensation by phosphorylating histone H3 [15]. When it is with the centromere, Aurora B phosphorylates the mitotic centromere-associated kinesin MCAK and regulates its localization and microtubule depolymerization activity [16], [17]. In yeast, it also phosphorylates the Dam1 outer-kinetochore microtubule-binding ring and the Ndc80 complex to facilitate formation of bipolar connections between the chromosomes and the spindle [18]. When located at the spindle midzone, Aurora B phosphorylates MKLP1/Pavarotti/ZEN-4 to establish the central spindle during anaphase [15], [19], [20]. Finally, Aurora B phosphorylates BimC for localizing BimC to the mitotic spindles in *Caenorhabdites elegans* [21].

We have recently identified an Aurora B ortholog in *T. brucei* (TbAUK1), and shown that it is required for spindle formation, chromosome segregation and cytokinesis in both procyclic and bloodstream *T. brucei* [11], [22]. TbAUK1 is localized to the nucleus in G2 phase and concentrated to the spindle midzone in late anaphase [11], [22]. While this suggests that TbAUK1 is a typical chromosomal passenger protein, homologs of INCENP, Survivin, Borealin, MKLP1/Pavarotti/ZEN-4, BimC or the Dam1 and Ndc80 complexes are all missing from the trypanosome genome database [23] raising the question of how TbAUK1 regulates mitosis and cytokinesis in *T. brucei*.

Here we report the identification of four novel TbAUK1 associated proteins and show that they are required for spindle formation, chromosome segregation and cytokinesis in procyclic trypanosomes. TbKIN-A and TbKIN-B are divergent novel kinesins that localize to both the nucleus and spindle whereas the TbAUK1-TbCPC1-TbCPC2 complex moves like a CPC during mitosis, and localizes to the anterior tip of the daughter cell tethered to the dorsal mid-point of the mother during cytokinetic initiation and, subsequently, to the branching point between the two dividing cells constituting the tip of a potential cleavage furrow. This finding identifies the TbAUK1-TbCPC1-TbCPC2 complex as the trypanosome CPC with TbCPC1 and TbCPC2 bearing no structural similarity to INCENP, Borealin/Dasra-B or Survivin of other eukaryotes.

## Results

### Identification of TbAUK1-associated proteins

**Figure 1:**
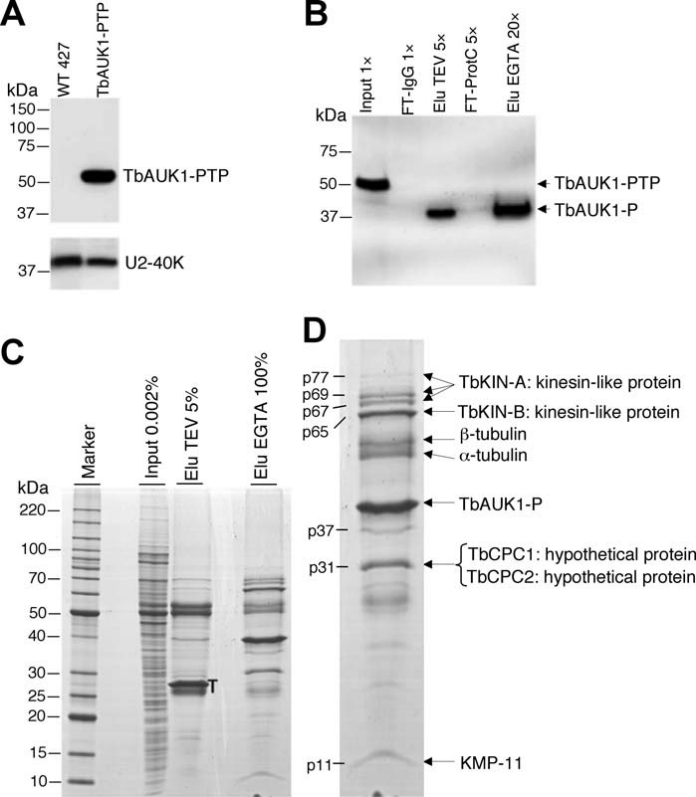
PTP purification and mass spectrometric identification of TbAUK1-associated proteins. (A) Whole lysates of WT 427 and TbAUK1-PTP cells were separated on a 10% SDS-polyacrylamide gel, blotted, and detected with the protein A-specific PAP reagent. The same blot was stained with a polyclonal antibody against U2-40K, a nuclear spliceosomal protein, as a loading control. (B) TbAUK1-PTP and TbAUK1-P following TEV protease digestion were detected with anti-ProtC antibody in the input material, IgG Sepharose column flow-through (FT-IgG), TEV protease-digested eluate (Elu TEV), anti-ProtC matrix flow-through (FT-ProtC), and final EGTA eluate (Elu EGTA). Values with x on top indicate relative amount of each fraction analyzed. (C) Coomassie staining of purified proteins. Elu EGTA was separated on a 10 to 20% SDS-polyacrylamide gradient gel and stained with Coomassie. For comparison, 0.002% of Input and 5% of Elu TEV were co-analyzed. T, TEV protease. (D). Mass spectrometric identification of the precipitated proteins. p77, p69, p67, etc stand for the estimated molecular masses of individual protein bands. On the left of panels A, B, and C, sizes of protein marker bands are indicated.

Since no close homologs of chromosomal passenger proteins other than Aurora B were identified in the *T. brucei* genome, we employed tandem affinity purification to isolate proteins that form complexes with TbAUK1 *in vivo* using the PTP (proteinA/tobacco etch virus protease site/proteinC epitope) tagging and purification system, which was recently established in *T. brucei* [24]. First, TbAUK1 was PTP-tagged at its C-terminus and expressed at the endogenous level in procyclic trypanosomes by targeted integration of the PTP vector into an endogenous *TbAUK1* allele (Fig. 1A). TbAUK1-PTP was then purified from a crude cell extract consecutively by IgG affinity chromatography and anti-proteinC epitope immunoaffinity chromatography. Both purification steps were highly efficient (Fig. 1B) and led to the detection of several proteins that were isolated with the TbAUK1-PTP fusion protein (Figs. 1C and 1D). Tryptic digest of each protein band was analyzed by liquid chromatography-tandem mass spectrometry (LC/MS/MS) which resulted in the identification of four novel proteins, designated TbKIN-A, TbKIN-B, TbCPC1 and TbCPC2 for reasons stated below (KIN stands for kinesin whereas CPC represents chromosomal passenger complex), and a fifth protein, which turned out to be the known membrane protein KMP-11 [25] (Fig. 1D). In addition, abundant α- and β-tubulins were co-purified, which could be attributed to the association of TbAUK1 with spindle microtubules [22]. Three protein bands (p77, p69 and p67), all identified as fragments of TbKIN-A, were considered degraded forms of TbKIN-A, which has a calculated molecular mass of 96 kDa. A p37 protein band was identified as either a degraded form of TbAUK1-PTP or the endogenous untagged TbAUK1. Other minor protein bands between p11 and p31 were all found to be degraded products of IgG light chain.

Among the five newly identified proteins, TbKIN-A (GeneDB accession number Tb11.02.0400) and TbKIN-B (Tb927.7.5040) are novel kinesin proteins that cannot be classified into any known kinesin groups [26]. They both possess the N-terminal kinesin motor domain and several coiled-coil motifs at their C-termini (Fig. S1A). The kinesin motor domain in TbKIN-A is ∼30% identical to that of BimC, MKLP1, CENP-E and MCAK, whereas that of TbKIN-B bears only ∼15% identity to them (Fig. S1B). TbKIN-B lacks several well-conserved and essential residues in the motor domain such as the lysine residue in the NTP-binding motif (Fig. S1B, arrow) and the SSRSH motif (Fig. S1B, Black line) raising the possibility that it may not be a functional kinesin.

TbCPC1 (Tb927.6.4820) and TbCPC2 (Tb11.01.6470), which were identified in two closely migrated protein bands p31 on the gel (Fig. 1D) and are similar in calculated molecular masses (∼28 kDa), have distinct sequences. They do not possess any known functional domains and exhibit no similarity to any protein with known function. They have no detectable sequence similarity to the chromosomal passenger proteins in other organisms. Both proteins are, however, well conserved among kinetoplastids (Figs. S2A and S2B). KMP-11 was previously reported to be a membrane protein and localized to flagellum and flagellar pocket [27], [28]. It is well conserved among the kinetoplastids [25] and bears no similarity to any known chromosomal passenger protein.

### TbAUK1 forms complexes with TbKIN-A, TbKIN-B, TbCPC1 and TbCPC2

**Figure 2:**
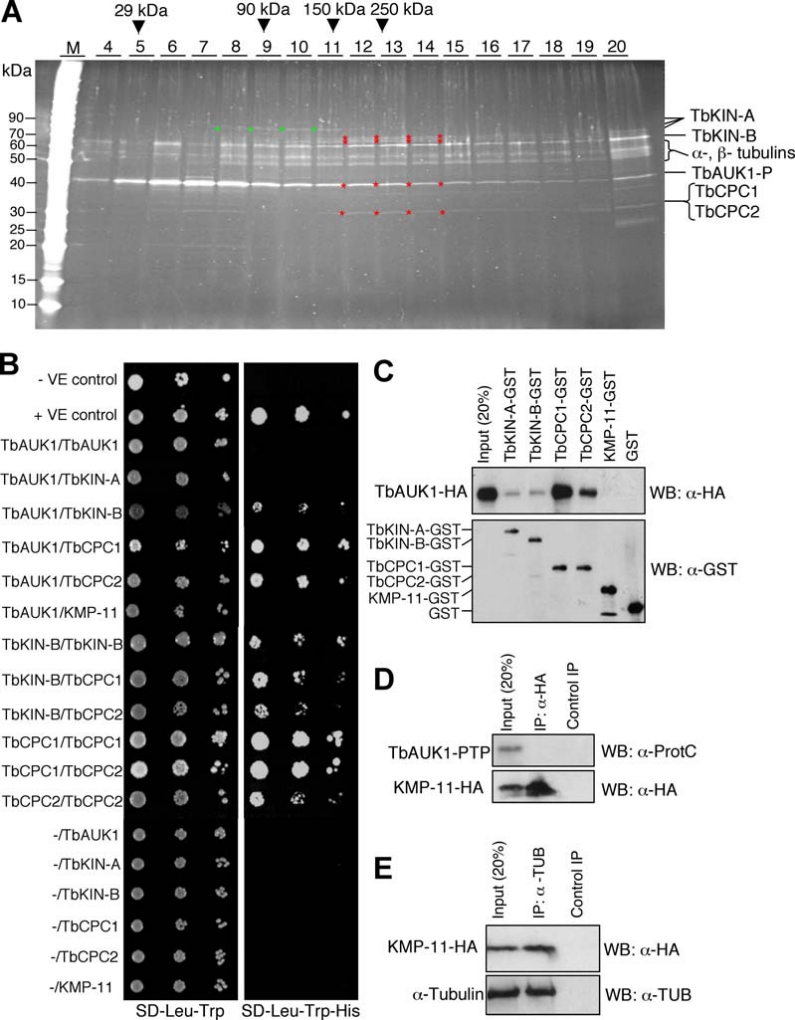
Interactions among TbAUK1 and TbKIN-A, TbKIN-B, TbCPC1 and TbCPC2. (A). Sucrose density gradient centrifugation of the purified TbAUK1-P complex. Red asterisks indicate the location of a complex among TbAUK1-P and TbKIN-A, TbKIN-B, TbCPC1 and TbCPC2. Yellow asterisks indicate the free forms of TbAUK1-P, TbKIN-B, TbCPC1 and TbCPC2. The green asterisks show the p77 fragment of TbKIN-A. (B). Yeast two-hybrid assay. Full-length TbAUK1, TbKIN-A, TbKIN-B, TbCPC1, TbCPC2 and KMP-11 were each cloned into the pGADT7 vector for expression of proteins fused to the Gal4 activation domain (prey) or into the pGBKT7 vector for expression of proteins fused to the Gal4 binding domain (bait), transformed to yeast strains AH109 and Y187, respectively. Each mated strain was then spotted onto SD-Leu-Trp and SD-His-Leu-Trp plates; with the latter selecting for interacting bait and prey proteins. (C). *In vitro* GST pull-down monitored on a Western with anti-HA mAb to detect TbAUK1-HA bound to the GST-fusion proteins. Loading of GST-fusion proteins as well as GST alone was monitored by a Western blot with anti-GST mAb. (D). Co-immunoprecipitation testing potential interaction between KMP-11-HA and TbAUK1. (E). Co-immunoprecipitation testing potential interaction between KMP-11-HA and α-tubulin.

To investigate whether the five proteins form one or more complexes with TbAUK1-P, final eluate of the PTP purification was spun in a sucrose gradient by ultracentrifugation. Subsequently, the gradient was fractionated and the proteins of each fraction collected, separated by denaturing SDS-PAGE, and detected by sypro ruby-staining (Fig. 2A). A large amount of TbAUK1-P, as well as some TbKIN-B, TbCPC1 and TbCPC2, were found in the free form in the gradient (Fig. 2A, fractions #6–8). TbKIN-A in the p77 form was detected in fractions #8–11. TbKIN-B (p65) appeared to be spread out in the entire gradient partly due to its association with the microtubules (data unpublished), but was enriched in fractions #12–15 where TbAUK1-P, TbKIN-A (p69 and p67), TbCPC1 (p31), and TbCPC2 (p31) were also identified (Fig. 2A, red asterisks). Fractions containing these five proteins correspond to protein complexes with a molecular mass of ∼250 kDa, which is similar to the calculated molecular mass of these five proteins combined (∼235 kDa). The five proteins are thus likely associated in a single complex.

KMP-11 was not detected in this sucrose gradient, which could be due to either a low abundance or a spread throughout the gradient due to interactions with many proteins. As the data in Fig. 2E will indicate, KMP-11 also binds to microtubules, which may have spread KMP-11 throughout the sucrose gradient.

To determine potential direct interactions among TbAUK1 and the other five proteins, we employed the yeast two-hybrid system, in which TbAUK1 interacted directly with TbKIN-B, TbCPC1 and TbCPC2, but not with TbKIN-A or KMP-11. Furthermore, TbKIN-B interacted with TbCPC1 and TbCPC2, and TbCPC1 with TbCPC2 (Fig. 2B). In contrast, the system did not reveal an interaction of TbKIN-A and KMP-11 with any of the other proteins (data not shown). TbKIN-B, TbCPC1, and TbCPC2, but not TbAUK1, formed dimers (Fig. 2B).

To confirm these findings, we performed *in vitro* GST pull down assays using purified recombinant GST-fusion proteins bound to glutathione Sepharose beads and the *in vitro* translated TbAUK1-HA. A Western blot with anti-HA antibody was then carried out to identify TbAUK1-HA bound to the GST-fusion protein. TbAUK1 was found to bind to TbKIN-A and TbKIN-B weakly but to TbCPC1 and TbCPC2 strongly whereas KMP-11 was not bound at all (Fig. 2C). Since none of these assays showed a direct interaction between TbAUK1 and KMP-11, a co-immunoprecipitation experiment was performed with TbAUK1-PTP and KMP-11-HA, which were both expressed in a procyclic cell line. The anti-HA antibody pulled down KMP-11-HA, but not TbAUK1-PTP (Fig. 2D). Instead, we found that KMP-11-HA was co-precipitated with α-tubulin (Fig 2E). We therefore concluded that KMP-11 was co-purified with TbAUK1 through association with microtubules and excluded it from further analysis.

### TbKIN-A and TbKIN-B localize to the nucleus and the spindle during mitosis and cytokinesis

**Figure 3:**
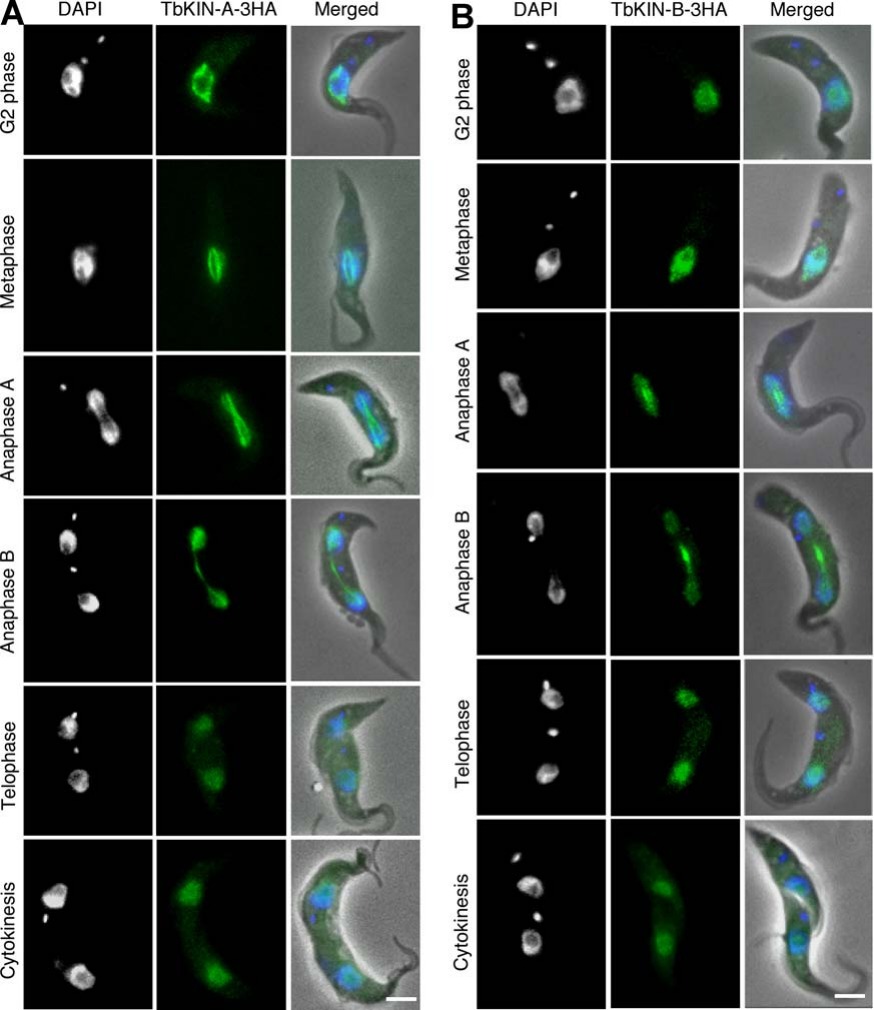
Subcellular localization of TbKIN-A and TbKIN-B in procyclic *T. brucei*. Cells expressing endogenous TbKIN-A-3HA (A) and TbKIN-B-3HA (B) were stained with FITC-conjugated anti-HA mAb, and counterstained with DAPI for DNA. Percentages of cells at different cell cycle stages were determined from about 900 cells in each sample. G2 phase (8% and 10% of the population for TbKIN-A and TbKIN-B, respectively); metaphase (7% and 6%); anaphase A (6% and 5%); anaphase B (9% and 10%); telophase (5% and 7%); cytokinesis (2% and 3%). The G1-phase (55% and 52%) and S-phase (7% and 8%) cells do not express the two proteins assayed by anti-HA immunofluorescence. Bars: 2 µm.

To determine the subcellular localization of TbKIN-A and TbKIN-B, we tagged each protein at the C-terminus with a triple HA epitope, expressed them in procyclic *T. brucei* at the endogenous levels, and examined them by immunofluorescence microscopy. TbKIN-A is primarily in the nucleus before the onset of mitosis, but it distributes to the entire spindle structure during metaphase and anaphase A. It remains associated with the spindle midzone in anaphase B but some of it is relocated to the two segregated nuclei and remains there during telophase and cytokinesis (Fig. 3A). Like TbKIN-A, TbKIN-B localizes to the nucleus in G2 phase, but enriched in the central spindle in anaphase A. In anaphase B, it is concentrated in the midzone and the two segregated nuclei and remains confined to the nucleus during telophase and cytokinesis. Neither TbKIN-A nor TbKIN-B ever moves outside of the nucleus (*T. brucei* has closed mitosis) (Fig. 3B). They are thus not typical chromosomal passenger proteins, which are known to trans-localize with Aurora B from the chromosomes to the midzone during anaphase B and telophase and to the cleavage furrow at the initiation of cytokinesis [13].

### TbCPC1, TbCPC2 and TbAUK1 exhibit an unusual localization pattern during mitosis and cytokinesis

**Figure 4:**
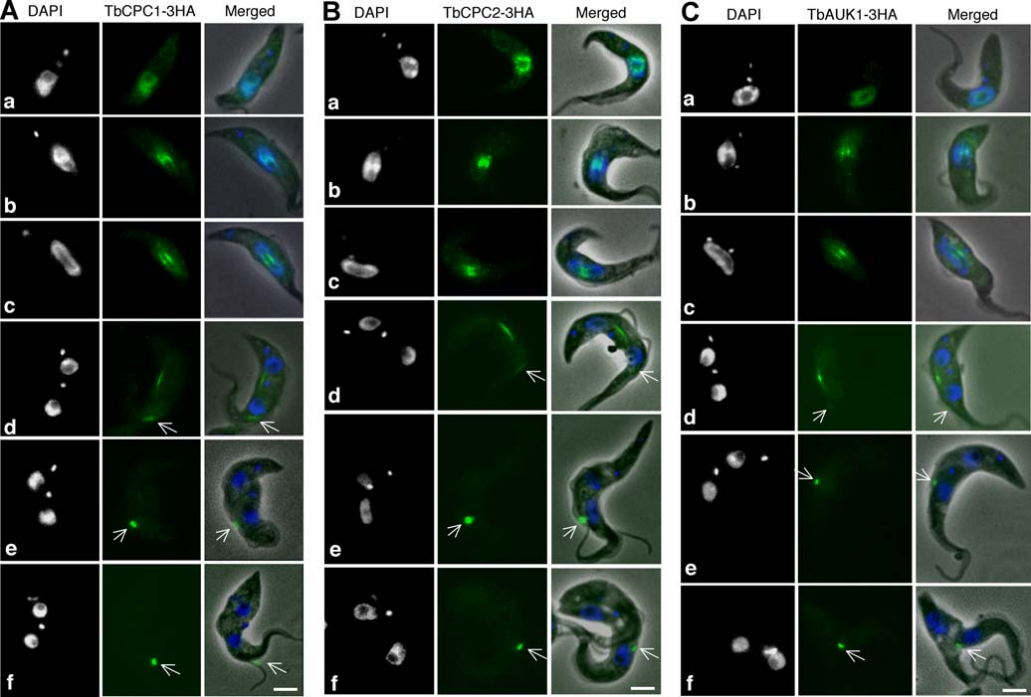
Subcellular localization of TbCPC1, TbCPC2 and TbAUK1 in procyclic *T. brucei.* Cells expressing endogenous TbCPC1-3HA (A), TbCPC2-3HA (B) and TbAUK1-3HA (C) were stained with FITC-conjugated anti-HA mAb, and counterstained with DAPI for DNA. a, G2 phase (8–10% of the population); b, metaphase (4–6%); c, anaphase A (6–8%); d, anaphase B (8–10%); e, telophase (4–6%); f, cytokinesis (1–3%). About 1,000 cells from each sample were examined and essentially all the cells were stained. The G1-phase (51–55%) and S-phase (9–12%) cells do not express these three proteins assayed by anti-HA immunofluorescence. Bars: 2 µm.

HA-tagged and endogenously expressed TbCPC1, TbCPC2 and TbAUK1 localize to the nucleus in G2 phase, appearing in the central spindle in metaphase and anaphase A, and concentrate in the spindle midzone in anaphase B (Figs. 4A, 4B and 4C, Panels a–d). Together with our biochemical data, these results identify TbCPC1, TbCPC2 and TbAUK1 as components of a tripartite CPC in *T. brucei.*

Most strikingly, in anaphase B, a portion of the protein complex started to show up at the mid-portion of the dorsal side of the cell in a bar-shaped distribution (Figs. 4A, 4B and 4C, Panel d, arrows). Then in telophase, the complex was completely localized to the dorsal side forming a well-defined spot, which could be closely associated with the anterior tip of the daughter cell (Figs. 4A, 4B and 4C, Panel e, arrows). This location is thought to define the initiation of cytokinesis in procyclic trypanosomes [5] and indeed, during cytokinesis, the three proteins remained at the dorsal spot except that it was now between the two split anterior ends of mother and daughter cells, which could constitute an unusual cleavage furrow during cell division (Figs. 4A, 4B and 4C, Panel f, arrows; see discussion below).

### RNAi silencing of the four genes led to similar mitotic and cytokinetic defects

**Figure 5:**
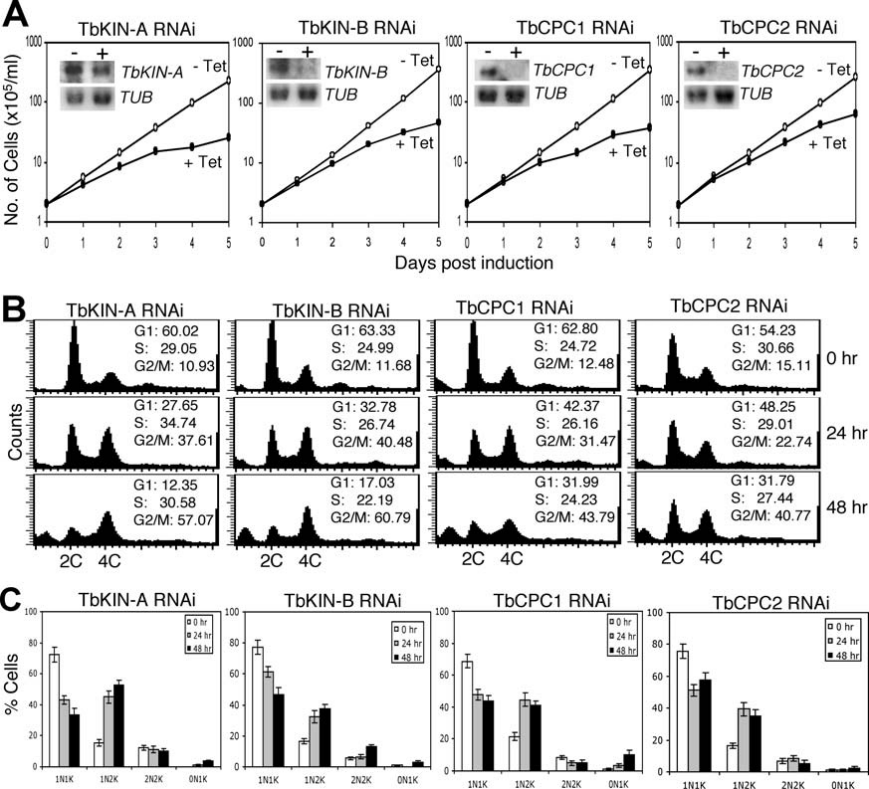
RNAi silencing of the four genes in procyclic *T. brucei.* (A). Clonal cell lines harboring the RNAi constructs were cultivated with (+Tet) or without (−Tet) tetracycline and monitored for cell growth. The insets show the levels of mRNA, monitored by Northern blot, in cells before (−) and after (+) RNAi for 2 days. α-Tubulin(*TUB*) was included as a loading control. (B). Flow cytometry analysis of DNA contents in RNAi cells. (C) RNAi cell lines after tetracycline induction for 0, 24 and 48 hrs were stained with DAPI and tabulated for numbers of nuclei (N) and kinetoplasts (K) in each cell. Data are presented as the mean percent±S.D. of ∼200 cells counted from three independent experiments. The experiment was repeated with three independent RNAi clones, and the results thus obtained were very similar to one another.

To evaluate the potential roles of the four proteins in cell cycle regulation, the phenotypic effects of their silencing in procyclic cells by an inducible RNAi system was analyzed. Northern blots showed, in each case, an appreciable reduction of the targeted mRNA two days after RNAi induction (Fig. 5A, insets), which coincided with an appreciable growth inhibition (Fig. 5A). Analysis by flow cytometry showed that a knockdown of each protein resulted in an accumulation of G2/M cells (4C DNA content), and a decrease in G1 cells (2C DNA content) (Fig 5B). A 2-day depletion of TbKIN-A, TbKIN-B or TbCPC1 each led to an increase of G2/M cells from ∼13% to ∼45–60%, whereas a knockdown of TbCPC2 increased G2/M cells from ∼15% to ∼40% (Fig. 5B). This outcome corresponds well to that from silencing TbAUK1 in a previous study [22].

**Figure 6:**
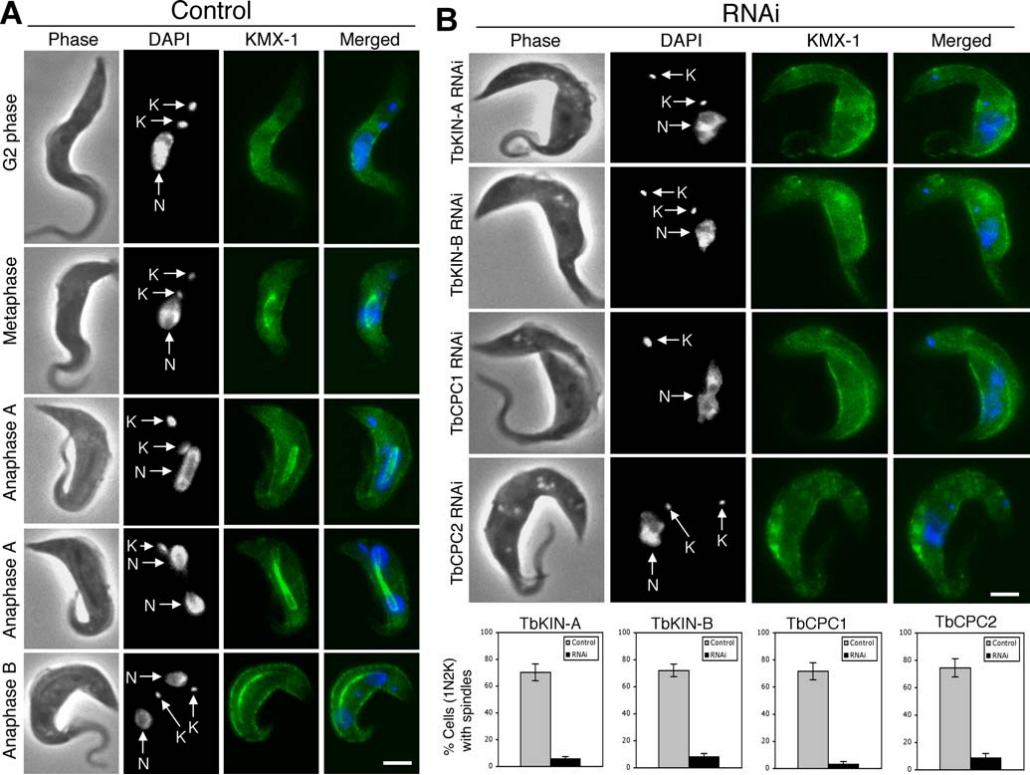
Effects of TbKIN-A, TbKIN-B, TbCPC1 and TbCPC2 knockdowns on spindle formation. Control (A) and RNAi (B) cells were stained with anti-β-tubulin mAb KMX-1 for spindle structures, and counterstained with DAPI for nucleus (N) and kinetoplasts (K). The 1N2K cells, enriched after RNAi induction, were examined. (C). Percentages of cells with visible spindle structures after 48 hr RNAi induction were presented as the mean percent±S.D. of ∼200 cells counted from three independent experiments. The experiment was repeated with three independent RNAi clones in each case, and similar results were obtained. Bars: 2 µm.

**Figure 7:**
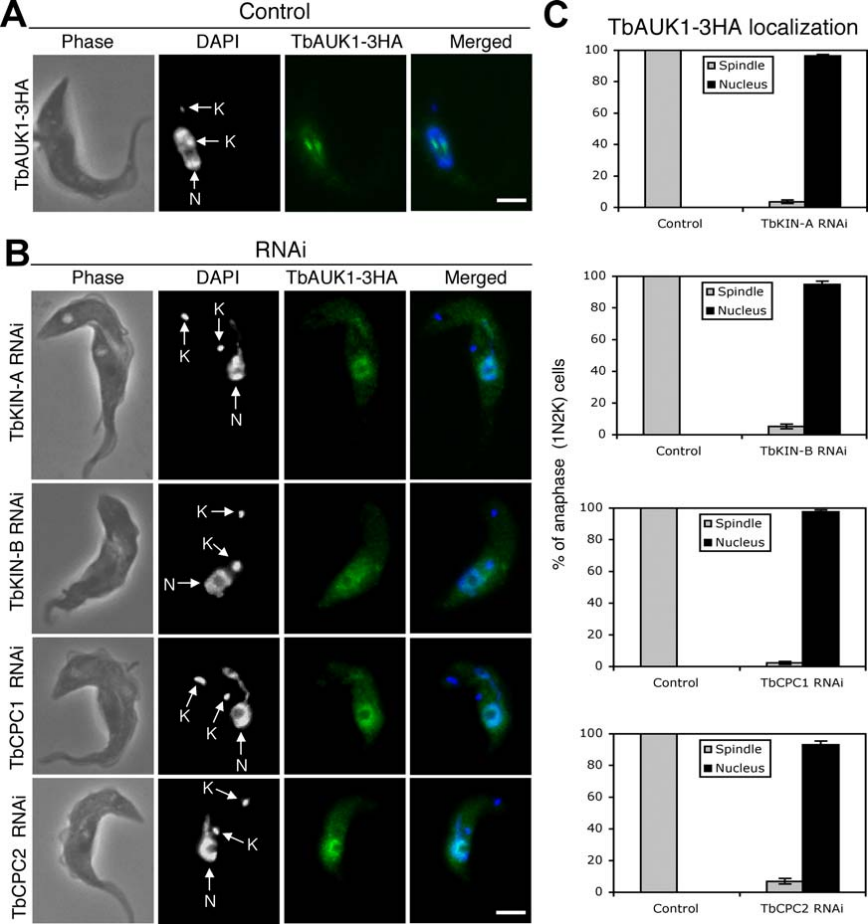
Effects of TbKIN-A, TbKIN-B, TbCPC1 and TbCPC2 knockdowns on subcellular localization of TbAUK1. (A). The endogenously expressed TbAUK1-3HA in un-induced control cell was detected with FITC-conjugated anti-HA mAb. (B). The endogenously expressed TbAUK-3HA in cells harboring TbKIN-A, TbKIN-B, TbCPC1 or TbCPC2 RNAi construct was detected with FITC-conjugated anti-HA mAb after a 48 hr RNAi induction. All stained cells were of the 1N2K type (N, nucleus; K, kinetoplast) in the anaphase stage of cell cycle. (C). Percentages of 1N2K cells in the anaphase stage with spindle or nuclear localization of TbAUK1-3HA in control and TbKIN-A, TbKIN-B, TbCPC1 and TbCPC2 RNAi cells. Data are presented as the mean percent±S.D. of ∼300 cells counted from three independent experiments. Bars: 2 µm.

To further characterize the potential mitotic and cytokinetic defects caused by depleting the four proteins, the RNAi cells were stained with DAPI for nuclear and mitochondrial DNA and the numbers of nuclei and kinetoplasts in each cell were counted. After a 48 hr RNAi induction, cells containing one nucleus and one kinetoplast (1N1K) were decreased significantly and accompanied by a corresponding increase in 1N2K cells from ∼15% to ∼40–50% with only minor changes in the numbers of 2N2K and anucleate cells (0N1K) (Fig. 5C). The 1N2K cells exhibited an elongated nucleus, indicating a late mitotic arrest, and two widely separated kinetoplasts, suggesting an arrest of cytokinesis after the segregation of two kinetoplast/basal body pairs (Fig. 6B). An outline of two closely associated cells with the daughter piggy backing on the mother was clearly demonstrated among all the 1N2K cells indicating again a cytokinetic arrest (Figs. 6B and 7). These phenotypes are very similar to those from knocking down TbAUK1 alone [22]. TbKIN-A, TbKIN-B, TbCPC1 and TbCPC2 are thus most likely acting together with TbAUK1 in promoting chromosome segregation and cytokinesis in *T. brucei*. A knockdown of any of the four proteins can result in a loss of TbAUK1 function leading to both mitotic inhibition and cytokinetic block after basal body segregation.

### RNAi silencing of TbKIN-A, TbKIN-B, TbCPC1 and TbCPC2 resulted in defect in spindle formation

Since TbAUK1 is essential for spindle assembly in *T. brucei* [11], [22], we examined whether the four partner proteins of TbAUK1 share this task. For this, un-induced and induced RNAi cells were stained with the anti-β-tubulin antibody KMX-1 for spindle structures [29], and the number of 1N2K cells with visible spindles was tabulated. While ∼70–80% of the 1N2K cells in the control possess a normal spindle structure from metaphase to late anaphase (Figs. 6A and 6C), a majority (>90%) of the RNAi induced 1N2K cells do not contain an identifiable spindle (Figs. 6B and 6C), suggesting that, like TbAUK1, TbKIN-A, TbKIN-B, TbCPC1 and TbCPC2 are needed for spindle formation.

### Silencing of TbKIN-A, TbKIN-B, TbCPC1 and TbCPC2 abolished the apparent trans-localization of TbAUK1

To analyze the effect of silencing the four proteins on the subcellular localization of TbAUK1, TbAUK1-HA was expressed in each of the four clonal RNAi cell lines. TbAUK1-HA expression was confirmed by Western blotting and the silencing of individual genes by Northern blotting (data not shown). Detection of TbAUK1 by immunofluorescence showed that when expression of TbKIN-A, TbKIN-B, TbCPC1 or TbCPC2 was silenced, the enriched 1N2K cell population (see Fig. 5C) had TbAUK1-HA expressed at a level comparable with the control indicating that a knockdown of each of the four genes did not affect the expression level of TbAUK1 (compare Figs. 4C, 7A and 7B). However, in each case, TbAUK1-HA stained diffusely in the nucleus and its apparent trans-localizations, as seen in the control cells, were not observed in over 90% of the 1N2K cells (Figs. 7B and 7C). This strongly indicates that each of the four proteins has an essential role in the apparent trans-localizations of TbAUK1 during mitosis and cytokinesis.

### Silencing of TbAUK1 affected the subcellular distribution of TbKIN-A, TbKIN-B, TbCPC1 and TbCPC2

**Figure 8:**
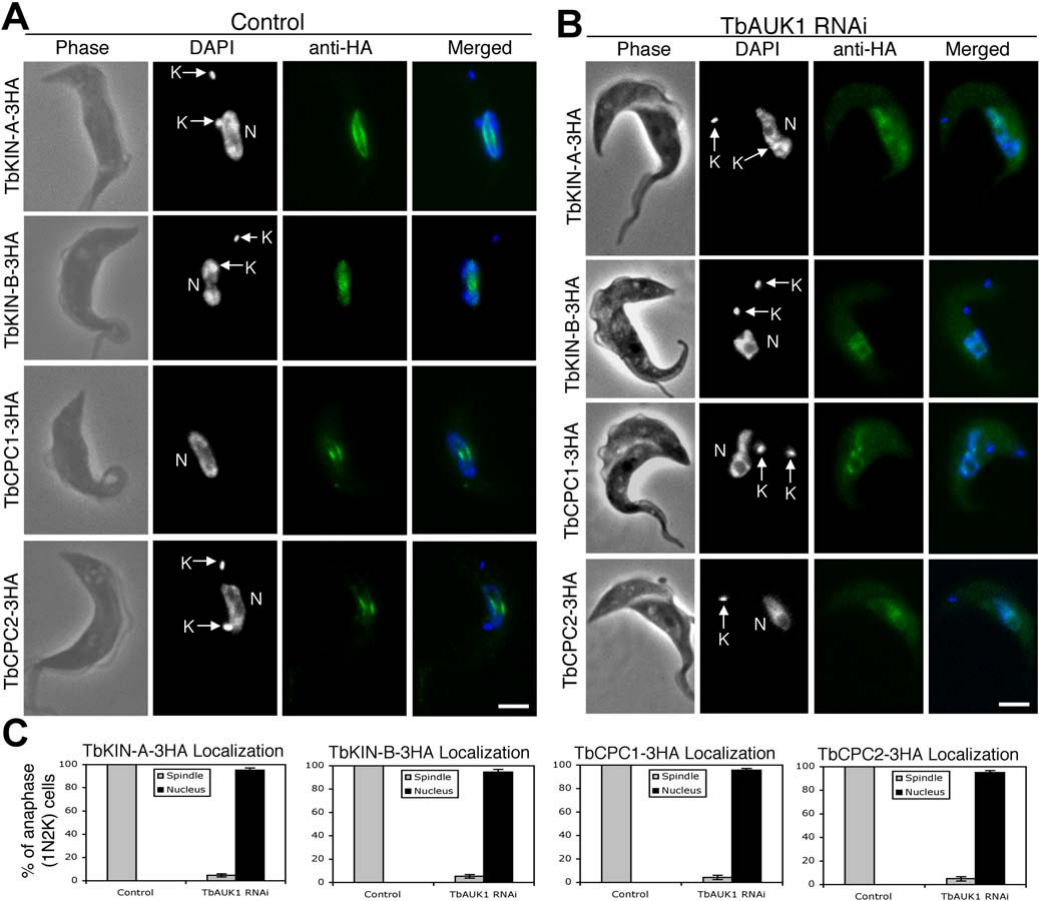
Effects of TbAUK1 knockdown on subcellular localization of TbKIN-A, TbKIN-B, TbCPC1 and TbCPC2. (A). The endogenously expressed TbKIN-A-3HA, TbKIN-B-3HA, TbCPC1-3HA and TbCPC2-3HA in un-induced control cells were detected with FITC-conjugated anti-HA mAb. (B). Localization of endogenously expressed 3HA-tagged TbKIN-A, TbKIN-B, TbCPC1 and TbCPC2 after a 48 hr RNAi of TbAUK1. All stained cells were of the 1N2K type (N, nucleus; K, kinetoplast) in the anaphase stage of cell cycle. (C). Percentages of 1N2K cells in the anaphase stage with spindle or nuclear localization of TbKIN-A-3HA, TbKIN-B-3HA, TbCPC1-3HA and TbCPC2-3HA in control and TbAUK1 RNAi cells. Data are presented as the mean percent±S.D. of ∼300 cells counted from three independent experiments. Bars: 2 µm.

In reciprocal experiments, we investigated how TbAUK1 silencing affected the subcellular distribution of TbKIN-A, TbKIN-B, TbCPC1 and TbCPC2. For this, each of the four proteins was HA-tagged and expressed endogenously in the TbAUK1 RNAi cell line. After confirming efficient knockdown of TbAUK1 mRNA by Northern blotting and expression of the HA-tagged proteins by Western blot (data not shown), each tagged protein was localized by anti-HA immunofluorescence in the 1N2K cells arrested before cytokinetic initiation. As shown in Figs. 8B and 8C, they were all confined to the nucleus in a diffused manner in over 90% of the 1N2K cells and their discrete changes of localization in the presence of TbAUK1 (see Figs. 3, 4A, 4B and 8A) could not be observed.

## Discussion

In this study, we identified four unique proteins that associate with TbAUK1. Two of them, TbKIN-A and TbKIN-B, are novel kinesins that trans-localize between chromosomes and spindle apparatus within the nucleus during mitosis. They are not the typical chromosomal passenger proteins, because they do not trans-localize with TbAUK1 throughout mitosis and cytokinesis. Although they are important for the apparent trans-localization of TbAUK1 (Fig. 7), they remain confined to the nucleus and become totally dissociated from the CPC when cytokinesis is initiated.

Kinesins constitute a super-family of microtubule-based motor proteins (for a review, see [30]). Several of them are required for mitosis [31]–[34], among which MCAK, BimC and MKLP1/Pavarotti/ZEN-4 are substrates of Aurora B in metazoans. Trypanosomes lack homologs of BimC and MKLP1/Pavarotti/ZEN-4, but possess a much-expanded MCAK family and many trypanosome-specific kinesins and highly divergent orphan kinesins [23], [26]. TbKIN-A and TbKIN-B are among the latter, and RNAi data suggested that they are essential for *T. brucei* cell division and may compensate for the absence of other well-known mitotic kinesins in this organism. The association of TbKIN-A with spindle microtubules in metaphase and anaphase A, and with the spindle midzone in anaphase B (Fig. 3A) resembles that of BimC [21], [35], [36]. But TbKIN-A localizes to the nucleus in trypanosomes, whereas metazoan BimC concentrates in centrosomes [21], [35]. In addition, RNAi of TbKIN-A abolished the trans-localization of TbAUK1 (Fig. 7), but localization of the Aurora B homolog AIR-2 in *C. elegans* is not dependent on the BimC homolog BMK-1 [21]. Thus, it is likely that TbKIN-A is functionally distinct from BimC.

The pattern of TbKIN-B trans-localization during mitosis (Fig. 3B) resembles that of *Drosophila* chromokinesin KLP3A [37] and MKLP1/Pavarotti/ZEN-4 [32], [34], [38]. However, unlike TbKIN-B, chromokinesin has not been found to interact with Aurora B and MKLP1/Pavarotti/ZEN-4 is not associated with chromatin and does not play a role in chromosome segregation. TbKIN-B may therefore play additional roles beyond those of the two kinesins.

The other two proteins, TbCPC1 and TbCPC2, appear to be the chromosomal passenger proteins closely associated with TbAUK1 throughout mitosis and cytokinesis. Although they are similar in size (∼28 kDa), they are not related to each other or to the known CPC components of yeasts and metazoans [39]–[43]. Assuming that the *T. brucei* CPC consists of TbAUK1, TbCPC1 and TbCPC2, it is smaller than metazoan CPC, which comprises four subunits as previously mentioned [43], but resembles the tripartite CPC of budding yeast with Ipl1p (Aurora kinase), Sli15p (INCENP) and Bir1p (Survivin) [44]. However, the molecular masses of Sli15p (79.2 kDa) and Bir1p (108.7 kDa) [39], [40] are much higher than those of TbCPC1 and TbCPC2.

Like the CPC components in human and *Drosophila*, TbAUK1, TbCPC1 and TbCPC2 are closely associated and mutually dependent in their locations during cell cycle progression in *T. brucei* (Figs. 7 and 8). This may explain why their individual knockdowns resulted in similar phenotypes, because knockdown of each protein delocalizes the others (Figs. 5, 6, 7 and 8). CPC subunits are essential for cytokinesis in metazoans, though detailed mechanisms are still unclear [13]. They are associated with the spindle midzone when cytokinesis is initiated. Ingression of the cleavage furrow, driven by a contracting actin/myosin II ring, closes onto the midzone to complete the process of cytokinesis [45], suggesting a limited migration of CPC away from the midzone, if at all. Therefore, the potential movement of the *T. brucei* CPC from the spindle midzone to the dorsal spot near the anterior tip of the daughter cell and then to the branching point between the anterior ends of mother and daughter during cytokinesis may constitute a highly unusual biological phenomenon, which, to our knowledge, has not been observed in any other living organism before (Fig. 4). The CPC in procyclic trypanosomes may have to dissociate from the spindle midzone in late anaphase, travel across the nuclear envelope to reach the dorsal mid-point near the anterior tip of the daughter cell where cytokinesis is to initiate [5]. This long and complex hypothetical journey of a CPC from the spindle midzone to a dorsal spot to initiate cytokinesis is unprecedented.

The components of cleavage furrow have yet to be identified in trypanosomes because an actomyosin contractile ring, which generates the furrow in yeasts and metazoans, is not found in *T. brucei* [6]. This actomyosin contractile ring is also not formed during plant cytokinesis [46]. But a preprophase band, which is a belt-like structure of actin filaments and microtubules, is formed at the cell cortex marking the cleavage furrow [46]. The furrow in trypanosome is probably drastically different from those in yeast, mammals or plants, because cytokinesis in trypanosome is initiated from the anterior end of the daughter cell located at the dorsal mid-point of the mother [5] and proceeds in a helical manner following the line of new flagellum/FAZ and cutting the cell in two [7]. The furrow is most likely associated with this line.

Following the duplication of the basal body/kinetoplast structure in procyclic *T. brucei*, a new flagellum is generated from the new basal body near the posterior end and grows alongside the axoneme of the old flagellum through a flagellum connector at the tip of new flagellum [47]. The outline of two cells with the daughter piggy-backing on the dorsal side of the mother with the tip of her anterior end tethered to mother's dorsal mid-point is formed prior to the completion of mitosis [47]. This form of double cells becomes more obvious when mitosis was arrested by knocking down the CPC (Fig. 6). The double anterior ends, formed upon initiation of cytokinesis, are not found without mitotic completion. We hypothesize that initiation of cytokinesis has to wait for the completion of mitosis and the subsequent change of localization of CPC from the spindle midzone to the mother's dorsal mid-point. The longitudinal cell division then commences with the CPC at the branching point, apparently spearheading the process toward the posterior end. In this scenario, the CPC would be both the initiator and the guide of cytokinesis in procyclic *T. brucei*.

In summation, we have identified the two novel TbAUK1-binding kinesin homologs TbKIN-A and TbKIN-B, which localize to the nucleus and spindle, and become dissociated from TbAUK1 in telophase. Moreover, we have characterized the *T. brucei* CPC, which contains TbAUK1 and the two novel proteins TbCPC1 and TbCPC2. Most interestingly, our data demonstrated that this unique CPC in telophase moves as a discrete spot to the dorsal mid-point of the mother cell where cytokinesis is initiated. The CPC then appears to move along with the longitudinal splitting of the cell indicating that it could be a regulator of this unusual mode of cell division. Hence, the CPC characterization in this study may provide us with a system for in-depth understanding of the mechanisms leading from mitotic exit to cytokinetic initiation and a possibility to study a different type of cytokinesis, which does not involve an actomyosin contractile ring.

## Materials and Methods

### Purification and analysis of the TbAUK1 complex

The C-terminal portion of TbAUK1 was cloned in-frame to the PTP-module in pC-PTP-NEO [24]. The resulting construct, pC-TbAUK1-PTP-NEO, was linearized at the unique *Nru* I restriction site, and transfected into the procyclic cell line 427. Transfectants were selected at 40 µg/ml G418 and cloned on an agarose plate [48]. Preparation of crude extract from 5×10^10^ procyclic cells, PTP purification, immunoblot monitoring of the purification, and analysis and detection of the purified proteins were performed exactly as described previously [24]. The ultracentrifugation analysis was carried out in a 4 ml 10–40% linear sucrose gradient as recently detailed [49]. Proteins of the gradient fractions were stained with SYPRO Ruby (Invitrogen, Carlsbad, CA) according to the manufacturer's protocol.

### In-gel Tryptic Digestion

In-gel digestion of individual protein bands was carried out utilizing a procedure described in http://msf.ucsf.edu/ingel.html. Typically, 100 ng of porcine trypsin (side chain-protected; Promega, Madison, WI) was used to digest each gel band at 37°C for 4 hr. Peptides were extracted from gel pieces with 50 µl of 50% acetonitrile, 2% acetic acid three times, and the extracts were combined and dried down to ∼10 µl.

### On-line Capillary LC-MS and LC-MS-MS Analysis

An 1 µl aliquot of the digest was injected into an Ultimate capillary LC system via a FAMOS Autosampler (LC Packings, Sunnyvale, CA), and separated by a 75 µm×15 cm reverse-phase capillary column at a flow rate of ∼330 nl/min. The eluent was connected directly to the micro-ion electrospray source of a QSTAR Pulsar QqTOF mass spectrometer (Applied Biosystem/MDS Sciex, Foster City, CA). Typical performance characteristics were >8000 resolution with 30 ppm mass measurement accuracy in both MS and CID mode. LC-MS data were acquired in an information-dependent acquisition mode, cycling between 1-s MS acquisition followed by 3-s low energy CID data acquisition. The centroided peak lists of the CID spectra were searched against the National Center for Biotechnology Information (NCBI) protein database using Batch-Tag, a program in the in-house version of the University of California San Francisco ProteinProspector package.

### RNA Interference

The procyclic form of *T. brucei* strain 29-13 [50] was cultured at 26°C in Cunningham's medium supplemented with 10% fetal bovine serum (Hyclone) and 15 µg/ml G418 and 50 µg/ml hygromycin B.

DNA fragments (300–500 bp) encoding the N-termini of the four genes were each cloned into the pZJM vector [51] for RNAi. Transfection of the procyclic form cells was performed as previously described [52]. The transfectants were selected under 2.5 µg/ml phleomycin and cloned. RNAi was induced by 1.0 µg/ml tetracycline. Cell growth was monitored by daily counting of cell numbers with a hemacytometer.

### Northern blotting

Total RNA was denatured and blotted onto nitrocellulose membranes. Northern blotting was performed as described previously [52]. The same blot was probed with α-tubulin fragment for equal loading of RNA samples.

### Flow Cytometry Analysis

The FACS analysis of propidium iodide (PI)-stained trypanosome cells was carried out as previously described [11]. The DNA content of PI-stained cells was analyzed with a fluorescence-activated cell sorting scan (FACScan) analytical flow cytometer (BD Biosciences). Percentages of cells in each phase of the cell cycle (G1, S, and G2/M) were determined by the ModFit LT V3.0 software (BD Biosciences).

### Epitope Tagging of Endogenously Expressed Proteins in T. brucei

C-termini of TbKIN-A, TbKIN-B, TbCPC1 and TbCPC2 were each cloned into the pC-3HA-Neo vector by replacing the PTP module in pC-PTP-Neo [24] with a triple HA epitope, and transfected into the 427 cell line. Stable transfectants were selected under 40 µg/ml G418. To tag the endogenous TbAUK1 in the four RNAi cell lines, TbAUK1 was cloned into the pC-3HA-Bla vector and transfected into the cells harboring the respective RNAi construct. For the reverse experiments, the four genes were each cloned into pC-3HA-Bla and transfected into the cell line harboring the pZJM-TbAUK1 RNAi construct. Stable transfectants were selected under 10 µg/ml Blasticidin.

### Immunofluorescence Assay

The following primary antibodies were used: KMX-1 for the spindles [29] and FITC-conjugated anti-HA mAb (Sigma-Aldrich) for HA-tagged proteins. Immunostaining was performed as previously described [53]. The slides were mounted in Vectashield mount medium and examined under a fluorescence microscope.

### Yeast Two Hybrid Assay

Full-length TbAUK1, TbKIN-A, TbKIN-B, TbCPC1, TbCPC2 and KMP-11 were each cloned into the pGADT7 vector for expression of proteins fused to the Gal4 activation domain (prey) or into the pGBKT7 vector for expression of proteins fused to the Gal4 binding domain (bait) (Clontech). Yeast strains AH109 (mating type a) and Y187 (mating type α) were transformed with the prey or the bait plasmid, respectively, and mated in YPDA media for 24 hrs at 30°C, followed by plating on SD-Leu-Trp plates for the presence of both plasmids. Each mated strain was then spotted in three ten-fold serial dilutions onto SD-Leu-Trp and SD-His-Leu-Trp plates; with the latter selecting for interacting bait and prey proteins.

### GST Pull-down

Full-length coding sequences of TbKIN-A, TbKIN-B, TbCPC1, TbCPC2 and KMP-11 were each cloned into a pGEM-4T-3 vector (Amersham), expressed in *Escherichia coli* BL21 cells and purified through glutathione Sepharose-4B beads. For GST pull-down experiments, TbAUK1-HA, produced from the TNT® *in vitro* transcription/translation system (Promega), was incubated with the purified bead-bound GST-TbKIN-A, GST-TbKIN-B, GST-TbCPC1, GST-TbCPC2, GST-KMP-11 or GST in the binding buffer and washed six times with the wash buffer [53]. The beads were then boiled in SDS sampling buffer, and the supernatant analyzed in SDS-PAGE followed by transfer to PVDF membrane and subsequent immunoblotting with anti-HA mAb and anti-GST mAb.

### Immunoprecipitation and Immunoblotting

Cells were incubated in the lysis buffer (25 mM Tris-Cl, pH 7.6, 100 mM NaCl, 1% Nonidet P-40, 1 mM dithiothreitol, and protease inhibitor cocktail) for 30 min on ice and cleared by centrifugation. The lysate was incubated with anti-HA mAb or anti-Tubulin mAb at 4°C for 1 hr and precipitated with protein G Sepharose beads overnight. The immuno-precipitates thus collected were fractionated in SDS-PAGE, transferred onto PVDF membrane and immuno-blotted with anti-Protein C mAb or anti-HA mAb as described [53].

## Supporting Information

Figure S1 Structural comparisons among TbKIN-A, TbKIN-B and the kinesins associated with Aurora B kinase in metazoans. (A). A schematic representation of different domains in TbKIN-A and TbKIN-B. The kinesin motor domain is shown in black and the predicted coiled-coil motifs are indicated by striped boxes. (B). Sequence alignment of the deduced amino acid sequences of the kinesin motor domains from TbKIN-A, TbKIN-B and the Aurora B kinase-associated kinesins in mammals. Identical and conserved residues are shaded in gray. The arrow points the conserved lysine residue in the NTP-binding motif and the black line indicates the conserved SSRSH motif.
(1.07 MB TIF)

Figure S2 TbCPC1 and TbCPC2 are well conserved among Kinetoplastids. Sequence alignment of TbCPC1 homologs (A) and TbCPC2 homologs (B) from *T. brucei*, *Trypanosoma cruzi* (GeneDB accession numbers: TcCPC1, Tc00.1047053506945.120; TcCPC2, Tc00.1047053506221.110) and *Leishmania major* (GeneDB accession numbers: LmCPC1, LmjF30.3480; LmCPC2, LmjF32.1640). Identical and conserved residues are shaded in gray.
(0.80 MB TIF)
