# Supplementary figures and images for: Identification of a Novel Chromosomal Passenger Complex and Its Unique Localization during Cytokinesis in Trypanosoma brucei
Source: PLoS One. 2008 Jun 11;3(6):e2354. doi: 10.1371/journal.pone.0002354 (PMC2396291; doi:10.1371/journal.pone.0002354)

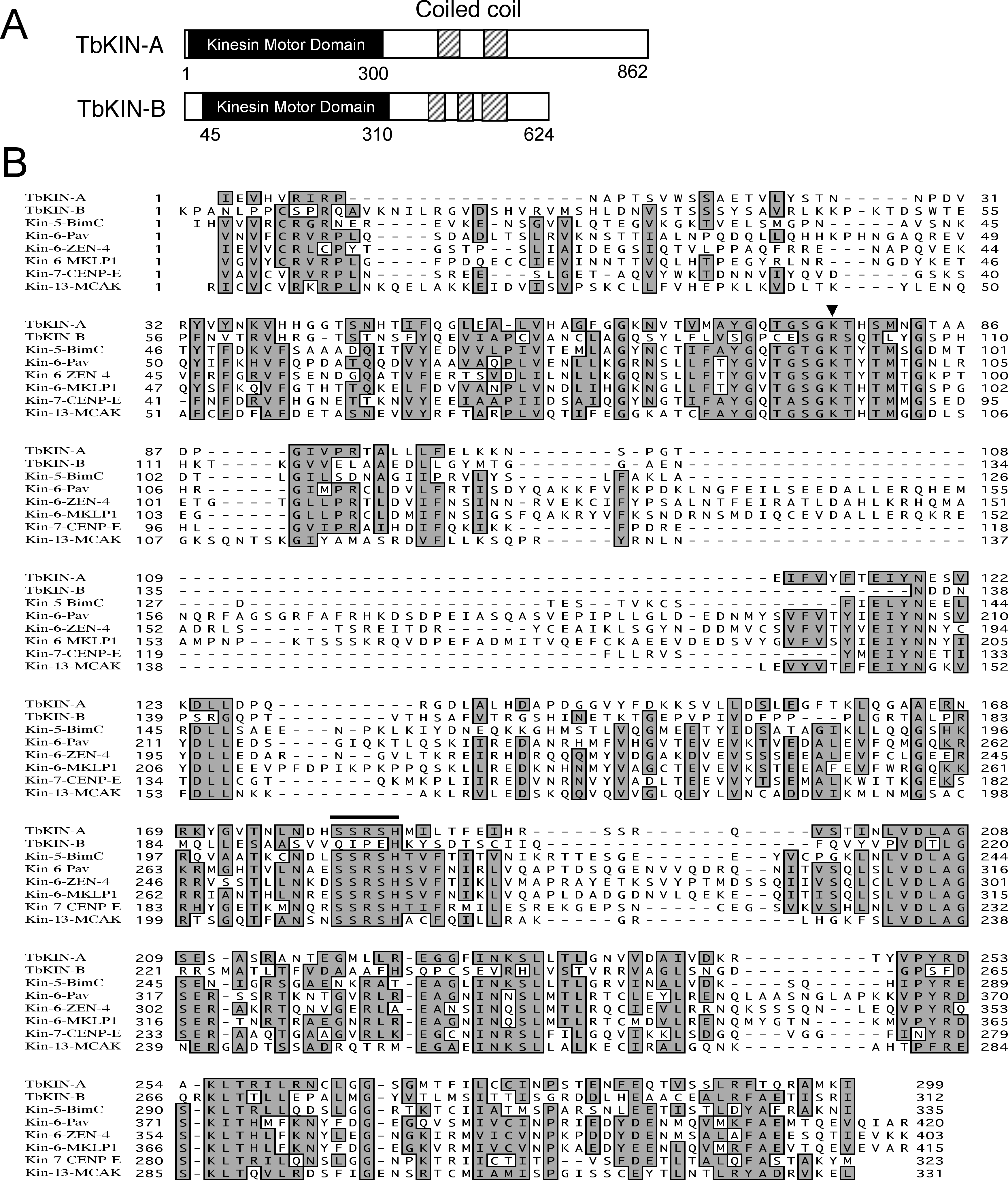

Supplement: Figure S1 — Structural comparisons among TbKIN-A, TbKIN-B and the kinesins associated with Aurora B kinase in metazoans. (A). A schematic representation of different domains in TbKIN-A and TbKIN-B. The kinesin motor domain is shown in black and the predicted coiled-coil motifs are indicated by striped boxes. (B). Sequence alignment of the deduced amino acid sequences of the kinesin motor domains from TbKIN-A, TbKIN-B and the Aurora B kinase-associated kinesins in mammals. Identical and conserved residues are shaded in gray. The arrow points the conserved lysine residue in the NTP-binding motif and the black line indicates the conserved SSRSH motif. (1.07 MB TIF) [file pone.0002354.s001.tif]

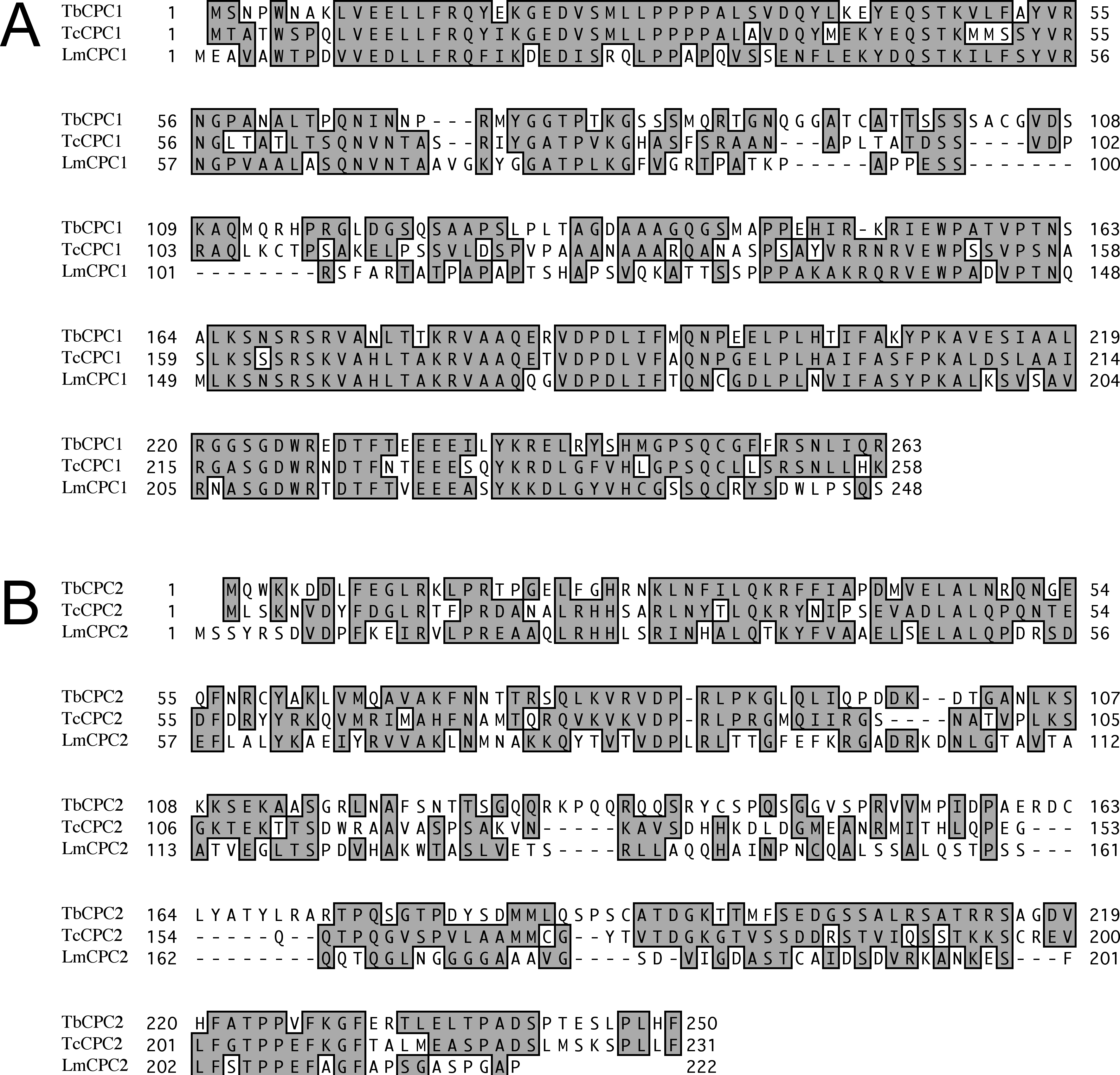

Supplement: Figure S2 — TbCPC1 and TbCPC2 are well conserved among Kinetoplastids. Sequence alignment of TbCPC1 homologs (A) and TbCPC2 homologs (B) from T. brucei, Trypanosoma cruzi (GeneDB accession numbers: TcCPC1, Tc00.1047053506945.120; TcCPC2, Tc00.1047053506221.110) and Leishmania major (GeneDB accession numbers: LmCPC1, LmjF30.3480; LmCPC2, LmjF32.1640). Identical and conserved residues are shaded in gray. (0.80 MB TIF) [file pone.0002354.s002.tif]
